# Aortic valvuloplasty under echocardiographic guidance in a minor infant at a national referral center in Peru: case report

**DOI:** 10.47487/apcyccv.v5i1.316

**Published:** 2024-03-19

**Authors:** Alex Catalán Cabrera, Karen Condori Alvino

**Affiliations:** 1 Área de Cateterismo Cardiaco Pediátrico, Instituto Nacional de Salud del Niño de San Borja, Lima, Peru. Área de Cateterismo Cardiaco Pediátrico Instituto Nacional de Salud del Niño de San Borja Lima Peru

**Keywords:** Aortic Valve Stenosis, Balloon Valvuloplasty, Heart Failure, Echocardiography, Cardiac Catheterization

## Abstract

Aortic valve stenosis is a congenital heart defect that causes a fixed left ventricular outflow obstruction with a progressive course. Symptomatology in neonates and young infants resembles congestive heart failure. In addition, the diagnosis of this condition is made by imaging, through echocardiography. On the other hand, treatment can be surgical or interventional under fluoroscopic guidance, depending on the hospital in which it is performed. We describe the case of a minor infant patient who presented severe aortic valve stenosis; however, the fluoroscopy equipment was not available at the time of the emergency to perform the appropriate procedure, therefore, an aortic valvuloplasty was performed under echocardiographic guidance without complications.

## Introduction

Aortic valve abnormalities represent 3.5 to 5% of all cardiac defects, where most cases show progressive worsening over time ^1^^,^^2^. According to biomedical literature, isolated critical aortic stenosis is uncommon and usually associated with other left-sided heart lesions, such as aortic coarctation, left ventricular hypoplasia, ventricular septal defects, and mitral valve pathology ^2^.

In neonates and younger infants, critical aortic valve stenosis presents with signs of low cardiac output and left ventricular dysfunction. In these cases, it is necessary to keep the ductus arteriosus open using prostaglandins, provide inotropic support to improve systemic perfusion, and, in most cases, respiratory support ^2^. Regarding echocardiographic evaluation, not only the gradient across the aortic valve but also left ventricular function should be assessed, and possible associated lesions should be ruled out. Critical aortic valve stenosis in newborns was associated with high morbidity and mortality in the past. However, it is currently associated with low morbidity and mortality, especially in centers where a higher volume of patients is attended ^3^. In fact, procedure-related mortality is reported in 3.0 to 4.5% of patients ^2^.

Interventional treatment should be performed in patients with normal cardiac output and a peak gradient greater than 75 mmHg and a mean gradient greater than 40 mmHg, without considering the gradient in patients with low cardiac output or severe left ventricular dysfunction ^4^. Specifically, the treatment of a patient with aortic valve stenosis can be carried out through surgery ^5^ or cardiac catheterization ^2^ under fluoroscopic guidance and, currently, under echocardiographic guidance ^6^.

Aortic valvuloplasty under echocardiographic guidance is a valid alternative and would have the advantage of avoiding the use of radiation and contrast agents ^6^, considering that patients in critical condition may have renal injury due to systemic hypoperfusion. Therefore, we present the case of a young infant patient who presented with severe aortic valve stenosis and underwent aortic valvuloplasty under echocardiographic guidance as a therapeutic option.

## Case Report

Male patient, 1 month and 24 days old, weighing 4 kg, born by normal delivery, non-syndromic, and without complications. The mother reports that one week prior, the infant experienced increased sweating and signs of respiratory distress, prompting a visit to a peripheral hospital and subsequently to our institution through the emergency department. Upon admission to our institution, he was admitted to the Intensive Care Unit with signs of low cardiac output, without a precise cardiovascular diagnosis.

**Figure 1:**
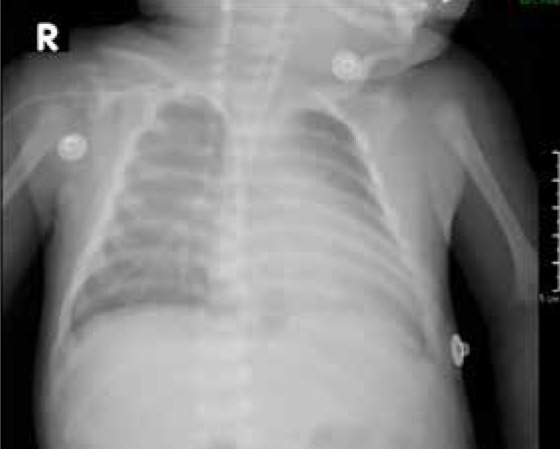
Pulmonary vascular congestion.

**Figure 2:**
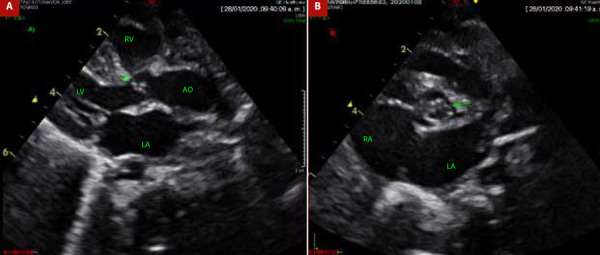
(A) Aortic valve morphology (B) Aortic annulus diameter.

**Figure 3:**
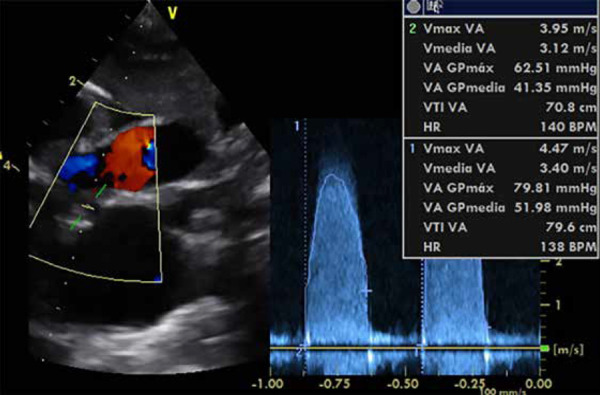
Aortic valve gradient.

As part of the auxiliary examinations, a chest X-ray was performed, showing signs of cardiomegaly with pulmonary vascular congestion (Figure 1). Likewise, an echocardiography was performed, revealing a bicuspid aortic valve with dysplastic leaflets, dome-shaped opening, with a Z score of -1 standard deviation for the aortic annulus, maximum systolic gradient across the aortic valve of 79 mmHg and mean gradient of 52 mmHg (Figures 2A, 2B, and 3). Additionally, the same examination revealed left ventricular dilation and hypertrophy, with reduced systolic function (left ventricular ejection fraction: 29%), without septal defects, without aortic coarctation, with normal systemic and pulmonary venous connections. Due to the emergency nature of the patient’s condition and having all findings defined by echocardiography, further diagnostic methods were not considered necessary.

LV: left ventricle. LA: left auricle. RA. right auricle. RV: right ventricle. AO: aorta

Through the auxiliary examinations performed, the diagnosis of severe aortic valve stenosis with reduced left ventricular systolic function was confirmed, findings that correlate with the degree of pulmonary compromise found on the chest X-ray. Over a short period of time (hours), the patient experienced clinical deterioration, prompting initiation of mechanical ventilation and inotropic support. At the time of the emergency, the hospital’s cineangiography team was not operational, and given the emergency, it was decided to perform aortic valvuloplasty under echocardiographic guidance. After evaluating the type and severity of aortic valve stenosis by echocardiography by the team composed of two pediatric cardiologists from the Hemodynamics Unit, an echocardiography cardiologist, and a pediatric anesthesiologist, it was decided to proceed with the procedure under echocardiographic guidance. 

The procedure was performed in the operating room with the patient under general anesthesia. Echocardiography was conducted using a General Electric S6 machine with an S6 transducer (GE Healthcare, Chicago, USA) with apical five-chamber, parasternal long-axis, and suprasternal views. Subsequently, 400 IU of unfractionated heparin was administered. The heart rate remained around 150 beats per minute; however, it was decided not to use a pacemaker considering the patient’s heart rate and the lack of availability of this device in the emergency situation.

**Figure 4:**
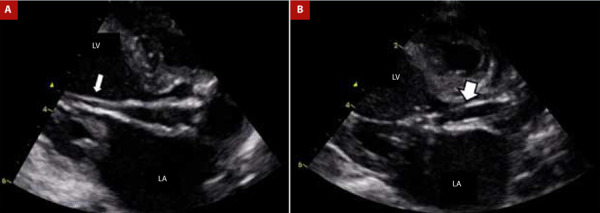
(A) 0.014” guide wire in the left ventricle through the aortic valve (thin white arrow). (B) 6 x 20 mm balloon catheter at maximum inflation (thick white arrow).

Through the right femoral artery using a 4 Fr introducer, a 4 Fr right coronary catheter with a 0.035” x 180 cm hydrophilic guidewire was inserted, which allowed positioning of the catheter in the ascending aorta. Subsequently, the 0.035” x 180 cm hydrophilic guidewire was exchanged for a 0.014” x 182 cm intermediate support guidewire to cross the aortic valve and position it at the apical level of the left ventricle. Once the 0.014” x 182 cm guidewire was positioned at the apical level of the left ventricle, the 4 Fr right coronary catheter was removed, and a Mini Tyshak 6 x 20 mm balloon (NuMED, New York, USA) was inserted until it was positioned at the level of the aortic valve. Under echocardiographic guidance, the balloon was inflated using an insufflation syringe to 4 ATM twice for a duration of ten seconds. Finally, the patient tolerated the procedure without complications (Figures 4A and 4B; Video 1).

LV: left ventricle. LA: left auricle

**Figure 5:**
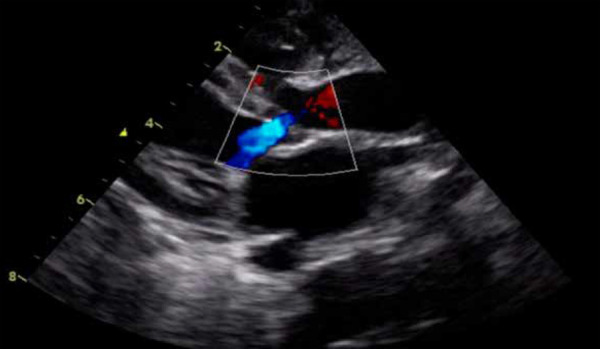
Mild aortic regurgitation after aortic valvuloplasty.

After the procedure, improvement in hemodynamic parameters is evident, while echocardiography showed progressive improvement in left ventricular systolic function, increased valve opening, with mild to moderate residual stenosis and mild aortic regurgitation (Figure 5).

The patient remained on mechanical ventilation for seven days, with gradual reduction of inotropic support, transferred to the general ward after four days of extubation, and discharged after three days. The patient has had serial outpatient follow-up appointments with clinical improvement and echocardiographic evaluations showing no increase in stenosis, with mild regurgitation, and no interventions in the last two years of follow-up.

## Discussion

In patients with aortic valve stenosis at early ages, each cardiac surgical group has adopted either surgery ^7^ or interventionism as the initial management method to relieve obstruction of the left ventricular outflow tract. Both procedures yield equivalent outcomes in the context of isolated valve stenosis ^8^^,^^9^.

According to biomedical literature, factors leading to an increased risk of reintervention include hypoplastic aortic ring, high post-procedure gradient, other associated left ventricular outflow tract obstruction lesions, aortic valve dysplasia, or the presence of a unicuspid aortic valve. However, aortic valvuloplasty ^7^ is considered to be safely performed with a significant impact on improving the patient’s immediate hemodynamic status.

In the presented case report, aortic valvuloplasty was performed under echocardiographic guidance due to the unavailability of an operational angiography system. This procedure has been previously reported by Li *et al.*^6^, where they used a catheter balloon-to-aortic annulus ratio of <1, similar to when performed under fluoroscopic guidance ^9^. Thus, the advantages of this procedure are associated with reduced radiation exposure and avoidance of contrast use.

In the reported case, the patient had compromised left ventricular function; however, the aortic valve gradient was severe, which was reduced to a mild degree post-valvuloplasty, resulting in improved left ventricular function and hemodynamic status. Additionally, aortic regurgitation in our patient was mild; although it is reported that it can be moderate to severe in up to 15-30% of cases ^5^.

Additionally, in our case, femoral artery access was used, but carotid and axillary access could also be used ^9^. Despite potential complications of femoral access occurring in up to 30-45% of cases, the patient did not experience any complications following this approach ^10^.

In conclusion, it should be noted that performing aortic valvuloplasty under echocardiographic guidance is feasible, especially when surgical options or angiography equipment are not readily available. Furthermore, this technique minimizes radiation exposure and avoids the use of contrast in a patient whose hemodynamic stability is already compromised.
